# Community- vs. healthcare-associated *Clostridium difficile* infections, Finland, 2008-2013: incidence, case fatality and genotypes

**DOI:** 10.1186/2047-2994-4-S1-P23

**Published:** 2015-06-16

**Authors:** SM Kotila, S Mentula, J Ollgren, A Virolainen-Julkunen, O Lyytikäinen

**Affiliations:** 1 National Institute for Health and Welfare, Helsinki, Finland

## Introduction

*Clostridium difficile* is not restricted to acute care hospitals.

## Objectives

The objective was to evaluate the incidence, case fatality and trends of community- (CA) vs. healthcare-associated (HA) *C. difficile* infections (CDI) in Finland during 2008-2013.

## Methods

CDI cases were identified from the National Infectious Disease Register to which all microbiology laboratories have notified toxin-positive *C. difficile* findings since 2008. Using cases’ national identity codes, dates of death were obtained from the National Population Information System and data of the National Hospital Discharge Register was used to classify cases as CA or HA. PCR ribotyping results were obtained from the reference laboratory.

## Results

A total of 33,303 CDI cases were identified; 10,874 (32.7%) were CA (33.7/100,000). The average annualised incidence rate of CA-CDI was significantly higher among persons aged 0-44 years, whereas in older age groups HA-CDI rates were higher. CA-CDI rate was higher in females than in males, especially in persons aged 15-44 years. The overall annual incidence rate of CDI decreased significantly, from 120.0/100,000 in 2008 to 93.4 in 2013, related to the decreasing rate of HA-CDI. 30-day case fatality was lower in CA-CDI than in HA-CDI in all age groups. Altogether 1211 *C. difficile* isolates were linked with CDI cases (3.6%), of which 268 were CA and 943 HA. In both groups the most frequent PCR ribotype was 027.

## Conclusion

While HA-CDI rate decreased, likely in response to improved infection control and increased awareness, CA-CDI rate remained stable. Preventive efforts, such as antimicrobial stewardship campaigns, should also cover long-term care and out-patient settings.

## Disclosure of interest

None declared.

